# Efficient and optimized blood cancer detection using engineered graphene-based silicon–TiN–silicon multilayered plasmonic sensor design with behaviour prediction using machine learning

**DOI:** 10.1038/s41598-025-33885-9

**Published:** 2026-01-03

**Authors:** Ammar Armghan, Yogesh Sharma, Aymen Flah, Meshari Alsharari, Khaled Aliqab

**Affiliations:** 1 Department of Electrical Engineering, College of Engineering, Jouf University, Sakaka, 72388 Saudi Arabia; 2 Department of Physics & Environmental Sciences, Sharda School of Engineering & Science, Sharda University, Greater Noida, Uttar Pradesh 201310 India; 3 Applied Science Research Center, Applied Science Private University, Amman, 11931 Jordan; 4 ENET Centre, CEET, VSB-Technical University of Ostrava, Ostrava, Czech Republic; 5 College of Engineering, University of Business and Technology (UBT), Jeddah, 21448 Saudi Arabia

**Keywords:** Optimization, Graphene, Plasmonic, Biosensor, Cancer, Efficient, Machine learning, Cancer, Engineering, Nanoscience and technology, Optics and photonics

## Abstract

Blood cancer can be fatal if not detected early; innovative biosensors with machine learning optimization enable timely diagnosis by identifying cancer-specific biomarkers in blood, improving survival rates through earlier intervention and targeted treatment. A graphene-based sensor, crafted with advanced materials, enhances sensitivity for rapid and early blood cancer detection, offering improved diagnostic accuracy and timely medical intervention for better patient outcomes. Machine learning optimization is used to achieve higher sensitivity. The graphene sensor achieves a maximum sensitivity of 1430 nm/RIU, enabling highly accurate and efficient blood cancer detection performance. The developed sensor demonstrates an impressive detection limit of 0.044, offering exceptional precision and sensitivity, making it highly effective for early-stage blood cancer diagnosis and clinical applications. The optimized sensor design achieves a high-quality factor of 125 and a figure of merit of 121, indicating excellent performance, sharp resonance, and enhanced precision for blood cancer detection applications. Optimization is achieved using parametric optimization. Optimization of the sensor is accomplished through detailed parametric analysis, resulting in a finely tuned design. This optimized structure significantly enhances sensitivity and detection speed, making it a highly suitable choice for early-stage and rapid blood cancer diagnosis, thereby improving the chances of timely treatment and patient survival.

## Introduction

Blood cancer is a deadly illness which involves the abnormal growth of blood cells, impacting vital component. This type of cancer includes various forms like leukemia, lymphoma, and myeloma, each with its own distinct characteristics and challenges. Effective analysis and diagnosis can reduce fatalities, as blood cancers can progress rapidly without warning. Without proper attention, the disease can lead to complications such as organ failure, weakened immune function, and increased risk of infections^1^. Diagnosing blood cancer requires a combination of advanced techniques. Early-stage symptoms can be subtle, often resembling other conditions, making it difficult to diagnose without proper medical attention. Therefore, healthcare professionals need to maintain a high level of vigilance when evaluating patient symptoms and risk factors^2^. Innovative diagnostic technologies, such as biosensors, are enhancing the sensor performance. These tools offer increased sensitivity, allowing for quicker identification of cancer-specific biomarkers in blood samples. Early detection facilitates prompt treatment interventions, including chemotherapy, radiation therapy, or stem cell transplants, improving the likelihood of successful outcomes and better quality of life for patients^3^. Machine learning models assist and help in early diagnosis of blood cancer. Predictive algorithms analyze both existing and new datasets to accurately identify cancer. These intelligent systems enhance diagnostic processes, enabling timely intervention and improving results through data-driven medical insights^4,5^.

Blood cancer detection using biosensors is an innovative and promising approach that leverages the sensitivity of advanced materials and detection technologies to identify specific biomarkers associated with blood cancers like leukemia, lymphoma, and myeloma^6^. Molecular or cellular makeup of blood samples are diagnosed, offering a non-invasive and rapid means of diagnosis. These sensors work by recognizing cancer-related biomarkers, such as proteins, DNA/RNA mutations, or abnormal cells, and providing accurate, real-time data for early diagnosis^7^. Biosensors are essential tools for the early identification of numerous malignant cells. These instruments detect unique biomarkers, offering swift, precise, and low-impact diagnostic results. Due to their remarkable accuracy and responsiveness, biosensors support cancer diagnostics, aid in timely medical response^8^.

Different types of biosensors are being developed to detect blood cancer. Electrochemical biosensors utilize conductive materials to measure changes in electrical signals when cancer biomarkers bind to the sensor surface. Optical biosensors detect shifts in light patterns or fluorescence signals caused by the presence of specific cancer-related molecules^9^. Graphene-having good sensitivity, allowing for highly accurate and rapid detection of minute amounts of blood cancer biomarkers^10^. Additionally, piezoelectric biosensors can detect changes in mass or frequency when cancer-specific particles interact with the sensor’s surface^11^. These biosensors significantly improve the speed and accuracy of blood cancer detection, offering a potential solution for early diagnosis, faster intervention, and better patient outcomes by facilitating timely treatment decisions. This research introduces cutting-edge techniques and clinical strategies for cancer detection, emphasizing novel diagnostic innovations. It explores emerging technologies, advanced screening methods, and their integration into clinical practice. The study highlights improved accuracy, early diagnosis, and potential for personalized approaches, contributing significantly to oncology diagnostics^12^.

Graphene-based sensors are highly promising designs for diagnosing blood cancers due to their exceptional physical, chemical, and electrical properties^13^. These biosensors utilize graphene’s extraordinary conductivity, high surface area, and biocompatibility to enhance the sensitivity and selectivity of cancer biomarker detection in blood samples. By facilitating the rapid and accurate identification of leukemia- and lymphoma-associated biomarkers, graphene biosensors significantly improve early cancer diagnosis, which is crucial for effective treatment planning and patient survival. Graphene sensors^14^. Graphene suspensions are employed to enhance sensing capabilities by increasing sensitivity and conductivity. Their uniform dispersion allows for better interaction with target analytes, leading to more accurate, rapid, and efficient detection in various biosensing applications, including cancer diagnostics^15^. Unlike traditional diagnostic methods, which often require complex procedures, high costs, and extended processing times, graphene-based biosensors enable real-time monitoring and point-of-care testing with minimal sample volume. Their functionalization flexibility allows the attachment of antibodies, aptamers, or DNA probes, which target specific cancer-related molecules such as proteins, enzymes, or genetic mutations^16^. Graphene oxides helping in improving the sensitivity of the sensor design^17^. Additionally, their integration with microfluidic systems and electronic platforms opens new avenues for the development of portable, user-friendly diagnostic devices. These innovative sensors can also be adapted for multiplex detection, allowing simultaneous monitoring of several biomarkers, thereby increasing diagnostic accuracy and efficiency. As research progresses, graphene biosensors are expected to become an integral part of personalized medicine, offering non-invasive, rapid, and highly accurate blood cancer diagnostics^18,19^.

Metamaterials are important in designing of biosensor because of effective properties^20^. These advanced materials have shown significant potential in the biomedical field, particularly in the early and rapid detection of cancer. Due to their remarkable ability to manipulate electromagnetic waves, metamaterials enhance the sensitivity and specificity of diagnostic devices. They can be designed to interact with biological tissues at a microscopic level, allowing for the detection of subtle changes associated with the presence of cancerous cells^21^. By integrating metamaterials into biosensing platforms, researchers can develop highly responsive and efficient sensors capable of identifying cancer at its earliest stages^22^. These sensors can detect specific biomarkers or variations in tissue properties with greater precision, leading to quicker and more accurate diagnostics. This capability is crucial for improving patient outcomes, as early diagnosis often leads to more effective and less aggressive treatment options^23^. Furthermore, metamaterials contribute to the miniaturization of diagnostic devices, making them more portable and accessible for point-of-care applications^24^. Their adaptability and customizable properties make them suitable for detecting a wide range of cancers, including breast, prostate, and skin cancers. Overall, metamaterials represent a groundbreaking advancement in medical diagnostics, significantly boosting the performance of cancer detection technologies^25^. This study focuses on the fabrication and characterization of highly transparent ZnO films deposited on p-type Si (100) substrates using RF magnetron sputtering. It further investigates the temperature-dependent structural, optical, and electrical properties of (Au, Al)/ZnO/p-Si heterojunction Schottky diodes sintered at different temperatures^26^. This work presents the Gradient Potential Dependent Skin-Depth Theory (GPST) to explain resonant plasmon-assisted tunnelling in metal nanoparticles. Using a silver nanodisk dimer system, the theory demonstrates how gradient potentials in sub-nanometer gaps govern tunnelling behavior, validated through finite-difference time-domain simulations^27^. Nowadays, the scope of developing device or layered material which uses for terahertz application^28^, and has a hepta-band characteristics^29^ have become emerging field.

The research highlights the urgent need for a fast and efficient blood cancer detection sensor. To address this, a graphene-based multilayer structure has been proposed. This innovative design offers promising potential for accurate diagnostics. Detailed discussions on the sensor’s design and performance follow in the subsequent sections.

## Design and modelling

**Fig. 1:**
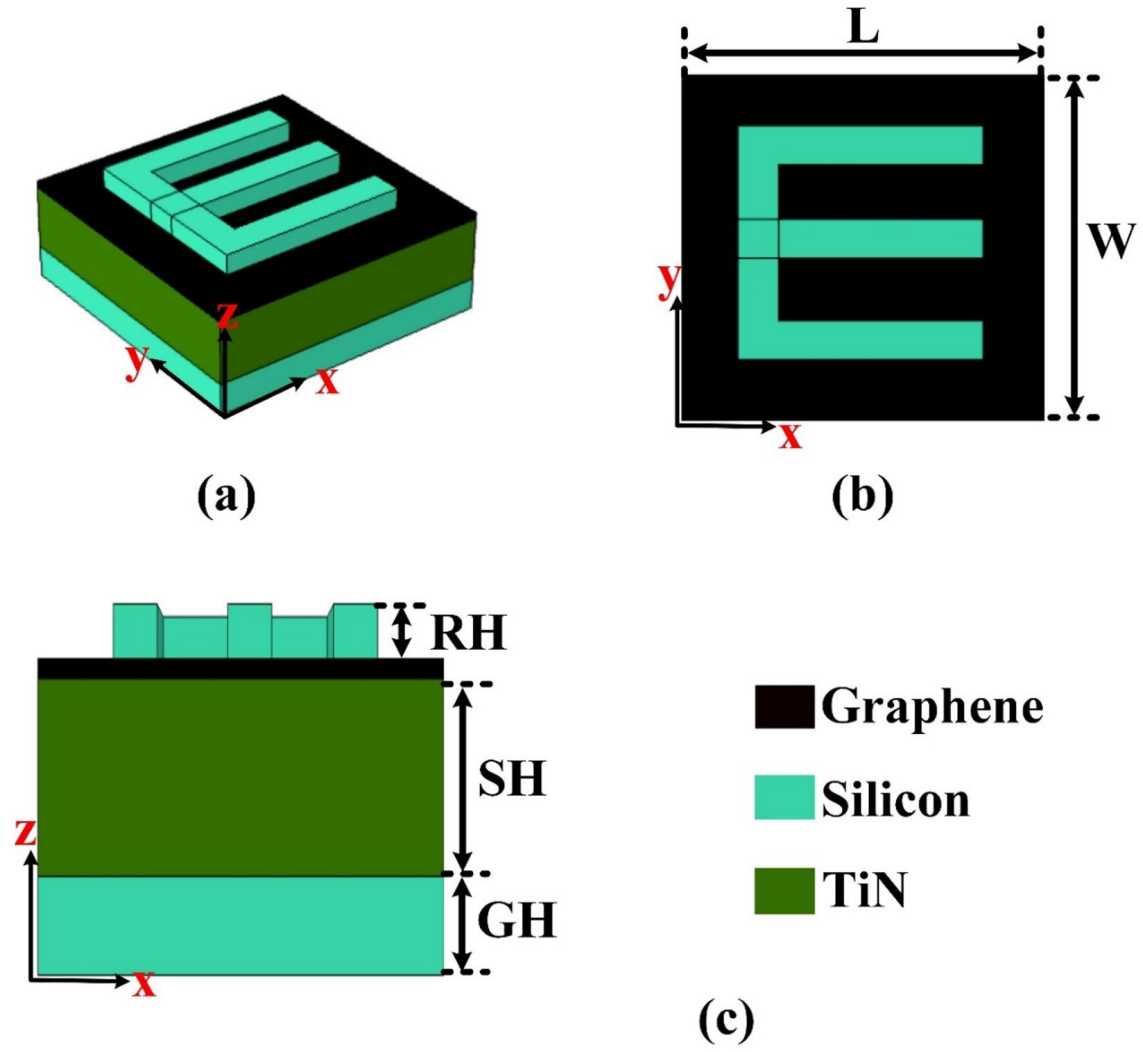
Sensor design and its views (**a**) E-shape 3D, (**b**) E-shape top view, (**c**) E-shape side view.

This section explores the multilayer design developed to enhance the sensitivity of blood cancer detection. The structure incorporates graphene which has a 0.34 nm thickness along with other complementary materials to achieve rapid and early diagnosis. Graphene’s exceptional electrical and optical properties contribute significantly to the sensor’s improved performance. By integrating multiple layers, the design optimizes signal interaction and detection accuracy. Figure 1 presents various perspectives of the proposed sensor, illustrating the configuration and spatial arrangement of each material layer. These visual representations aid in understanding how the design enhances overall functionality, making it a strong candidate for advanced, real-time biomedical diagnostics.

The multilayer structure employed in this study utilizes a combination of Silicon, Titanium Nitride (TiN), and additional Silicon layers which is shown in the layer by layer configuration. This carefully engineered arrangement is designed to optimize the sensor’s performance, particularly in enhancing its sensitivity for early detection of blood cancer. Silicon, known for its excellent semiconducting properties, provides a stable and efficient base and top layer, contributing to structural integrity and compatibility with existing fabrication technologies. Titanium Nitride (TiN), situated between the silicon layers, serves as a plasmonic material due to its desirable optical properties, thermal stability, and biocompatibility, which are crucial for bio-sensing applications.

By layering these materials in a precise configuration, the design ensures improved interaction between light and matter, thereby increasing the sensor’s ability to detect minute biological changes associated with the early stages of blood cancer. The multilayer structure allows for stronger electromagnetic field confinement and better signal-to-noise ratio, leading to more accurate and reliable readings. This novel combination of materials and architecture highlights the potential of the material approach in next-generation biosensor development, offering a promising path for rapid and effective cancer diagnostics.

**Fig. 2:**
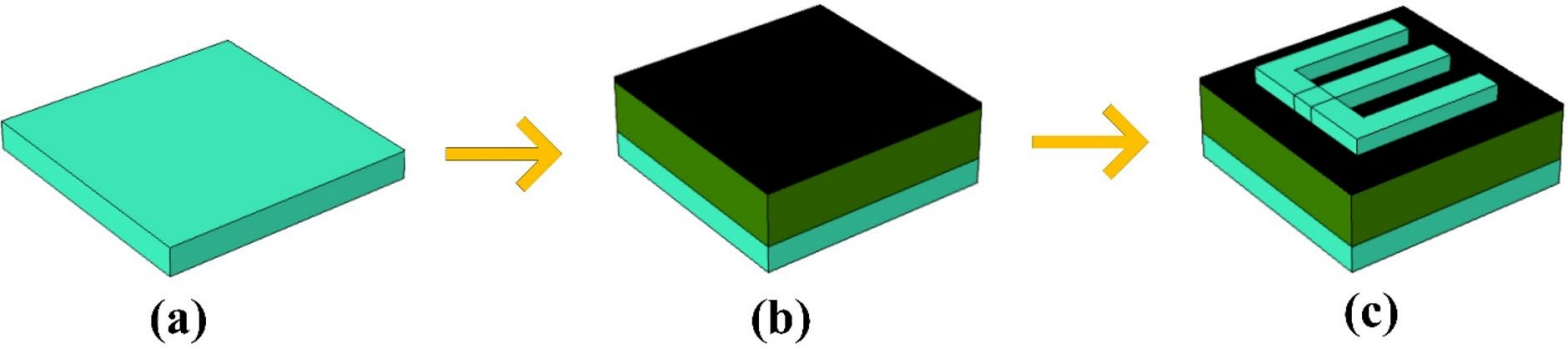
(**a–c**) Layered fabrication approach with deposition and etching.

The fabrication approach for the multilayer structure is a critical step in achieving the desired performance of the proposed blood cancer detection sensor. This process involves the precise deposition of multiple material layers, followed by patterning and etching to create the resonator structure. As illustrated in Fig. 2, the initial step involves depositing a base layer of silicon onto the substrate, which serves as the foundational platform. This is followed by the deposition of a Titanium Nitride (TiN) layer, selected for its excellent plasmonic properties, and then capped with an additional silicon layer to complete the multilayer configuration.

Once the multilayer stack is formed, advanced lithography techniques are used to define the resonator’s geometry. This involves applying a photoresist material, exposing it to a specific light pattern, and then developing the pattern to selectively remove areas of the resist. Subsequent etching processes remove the exposed material layers, creating the resonator with high precision. This combination of deposition and lithography ensures the creation of a highly accurate and repeatable structure. The fabrication strategy outlined in Fig. 2 not only ensures consistency in design but also enables scalable manufacturing, making it suitable for practical biomedical sensing applications.

Graphene is having hexagonal lattice, exhibits exceptional electrical properties that make it highly valuable in advanced electronic and sensing applications^30^. One of the key characteristics of graphene is its tunable conductivity, which varies significantly depending on its electrostatic potential. This dependence is fundamental to understanding and manipulating graphene-based devices^31^. The electrical conductivity of graphene is directly influenced by the carrier concentration, which in turn is controlled by the applied potential or gate voltage. When a voltage is applied, it shifts the Fermi level, thereby altering the density of charge carriers—either electrons or holes—within the material^32^. As the graphene potential changes, so does its conductivity, making it highly responsive to external electric fields. This relationship between graphene’s potential and its conductivity is mathematically described through a set of governing equations, typically represented in Eqs. (1–4)^33^. These equations provide a detailed model of how conductivity evolves with changes in electric potential, temperature, and other environmental conditions. Understanding these equations is crucial for optimizing the performance of graphene in sensors, transistors, and other nanoelectronic components, where precise control over conductivity is required for functionality. Hence, the interplay between potential and conductivity is central to graphene’s practical applications^34^.

1 $$\:\epsilon\:\:\left(\omega\:\right)=1+\:\frac{{\sigma\:}_{s}}{{\epsilon\:}_{0}\omega\:\nabla\:}$$

2 $$\:{\sigma\:}_{intra}=\:\frac{-j{e}^{2}{k}_{B}T}{\pi\:{\hslash}^{2}\:(\omega\:-j2\Gamma)}\left(\frac{{\mu\:}_{c}}{{k}_{B}T}+2\:\mathrm{ln}\left({e}^{\frac{{\mu\:}_{c}}{{k}_{B}T}}+1\right)\right)$$

3 $$\:{\sigma\:}_{inetr}=\:\frac{-j{e}^{2}}{4\pi\:\hslash\:}\mathrm{ln}\left(\frac{2\left|{\mu\:}_{c}\right|-(\omega\:-j2\Gamma)\hslash}{2\left|{\mu\:}_{c}\right|+(\omega\:-j2\Gamma)\hslash}\right)\:$$

4 $$\:{\sigma\:}_{s}={\sigma\:}_{intra}+{\sigma\:}_{inter}$$

Sensitivity (S) and its associated parameters play a critical role in evaluating the performance of sensors, particularly in advanced detection systems. The relationship between sensitivity and key performance indicators is quantitatively expressed in Eqs. (5–8). These equations describe how sensitivity interacts with essential metrics such as the Figure of Merit (FOM), Detection Limit (DL), and Quality Factor (Q-factor), which together define a sensor’s effectiveness and accuracy^35^. The Figure of Merit (FOM) is a comprehensive parameter that combines sensitivity with the resolution or sharpness of the sensor’s response, offering a clear indication of its overall performance. A higher FOM typically suggests better signal discrimination and more precise detection capability. The Detection Limit (DL), on the other hand, represents the smallest measurable signal or change that the sensor can reliably detect^36^. A lower DL implies higher sensitivity and more effective early detection, particularly important in applications like disease diagnosis or environmental monitoring. The Q-factor, or Quality Factor, reflects the sharpness of the resonance peak in optical or electrical sensing systems. A higher Q-factor indicates lower energy loss and more refined sensitivity. These interrelated parameters, when analyzed through Eqs. (5–8), provide a thorough understanding of a sensor’s capability, enabling enhanced design and application performance^37^.

5 $$\:S=\frac{\varDelta\:\lambda\:}{\varDelta\:n}$$

6 $$\:FOM\:=\:\frac{S}{FWHM}$$

7 $$\:Q=\frac{\lambda\:r}{FWHM}$$

8 $$\:DL=\:\left(\frac{\varDelta\:n}{1.5}\right)\times\:{\left(\frac{FWHM}{\varDelta\:\lambda\:}\right)}^{1.25}$$

## Results analysis

**Fig. 3:**
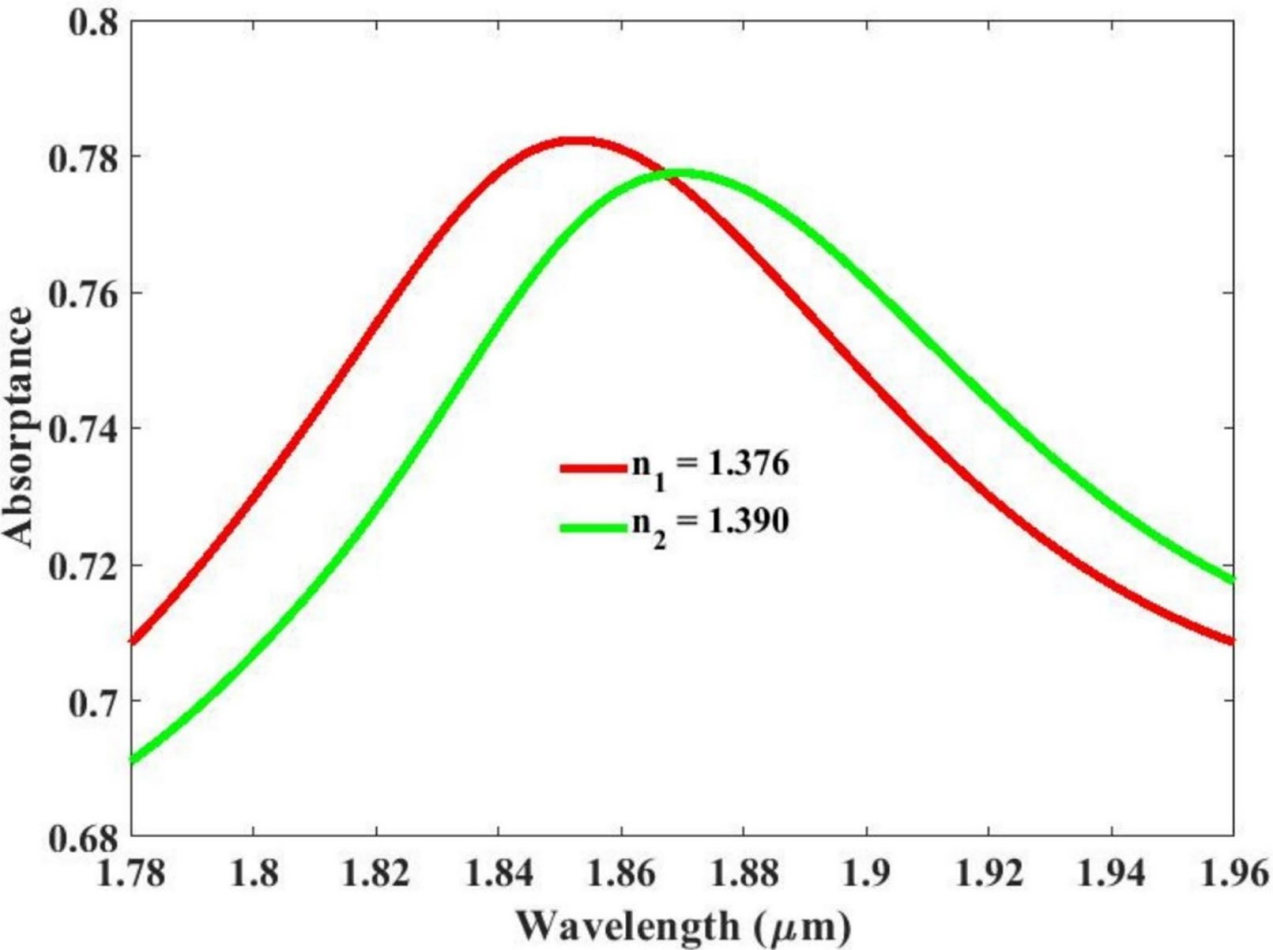
Sensor performance for the cancer cell and normal cell.

The structural analysis was performed utilizing the COMSOL Multiphysics software, and the corresponding simulation outcomes are illustrated in Fig. 3. This figure presents a comprehensive visualization of the computed results, reflecting the model’s behavior under periodic boundary conditions, tetrahedral meshing, and port excitation. Through finite element modeling in COMSOL, detailed insights into the structural performance, including stress distribution, deformation patterns, and other critical parameters, were obtained. The graphical representation in Fig. 3 highlights the areas of interest and provides a clear understanding of the simulation findings. These results serve as a foundation for evaluating the effectiveness and integrity of the analyzed structure. The sensitivity analysis is done using the Eqs. (5–8). The sensitivity of 1430 nm/RIU is achieved. In the current sensor configuration, the calculated Q-factor, FOM, and DL are found to be 125, 121, and 0.044 respectively. These values indicate the sensor’s performance in terms of sensitivity, resolution, and efficiency. A Q-factor of 26 signifies a moderate energy retention capability, implying sharp resonance characteristics. The FOM value of 21 reflects the sensor’s strong capability to distinguish small changes in the measured parameter, enhancing its precision. Meanwhile, a low DL value of 0.044 demonstrates the system’s ability to detect extremely small variations in the target stimulus. Collectively, these metrics confirm the proposed sensor’s potential for accurate, reliable, and highly sensitive detection applications across a variety of conditions.

**Fig. 4:**
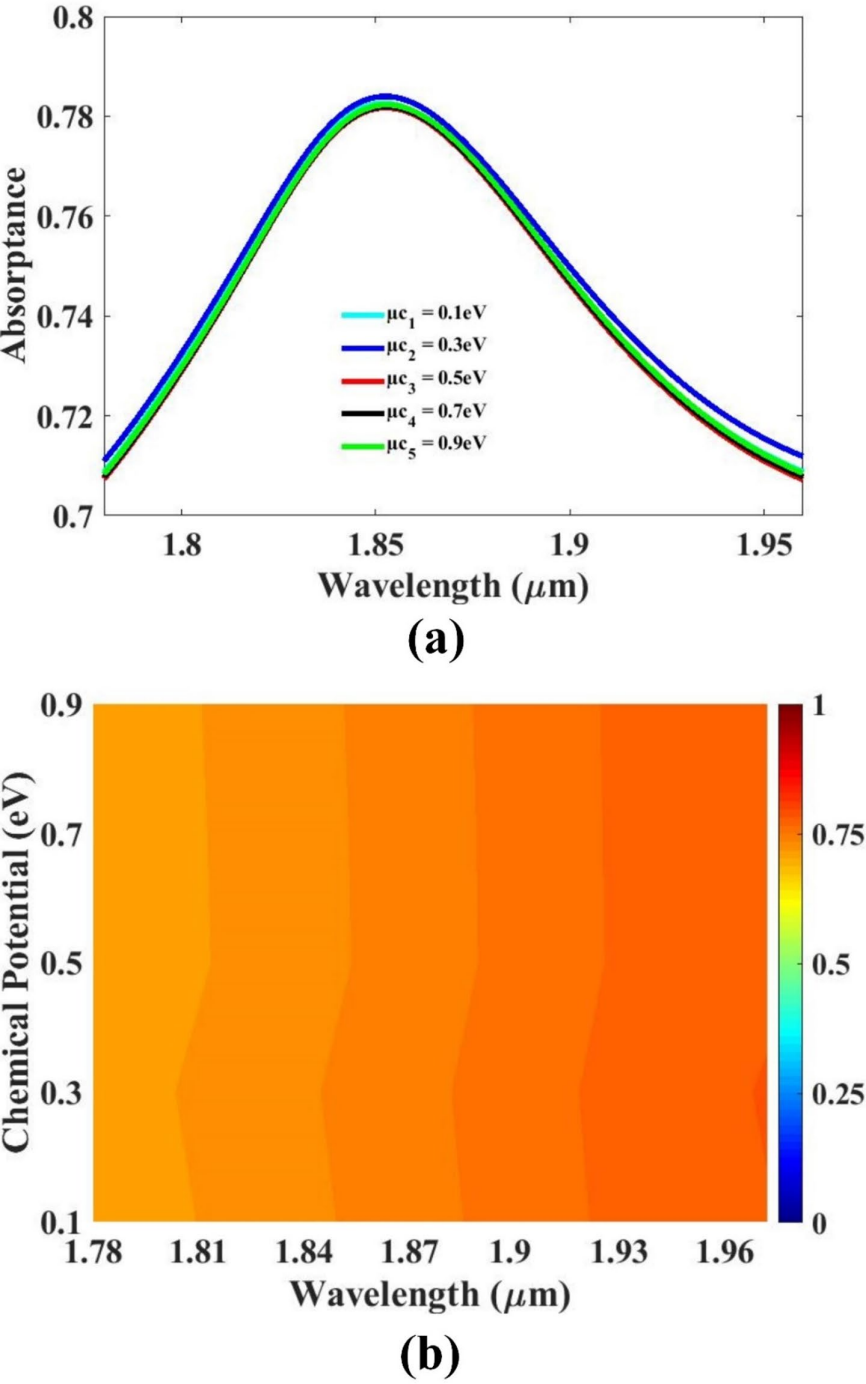
Graphene chemical potential variation plots (**a**) Line (**b**) color.

The electrical conductivity of graphene is intrinsically linked to its electrochemical potential, also known as the graphene potential. This potential plays a critical role in determining the material’s carrier concentration and type, directly influencing its conductive behavior. By varying the graphene potential through external stimuli such as gate voltage or chemical doping, the distribution of electrons and holes within the graphene lattice can be altered, thereby modulating its conductivity. This tunable nature of graphene’s electrical properties allows for dynamic control over the sensor’s operational characteristics. The results are visible in Fig. 4. As the graphene potential shifts, it causes noticeable changes in the electromagnetic response and sensitivity of the sensor. This phenomenon can be strategically exploited to fine-tune the sensor’s performance parameters, enabling real-time adaptability in diverse sensing environments. Such tunability is especially valuable in applications requiring high precision, responsiveness, and customization. Consequently, the ability to manipulate the graphene potential provides a powerful mechanism for optimizing sensor function, making graphene-based devices highly versatile and effective in a wide range of technological fields, including biomedical diagnostics, environmental monitoring, and optical communications. Overall, this sensitivity to potential variation underscores graphene’s role as a key material in the development of next-generation adaptive sensor systems.

### Parametric optimization

The use of optimized parameters significantly enhances the clarity and depth of analysis in sensor design, allowing for a more accurate evaluation of performance metrics and operational characteristics. These refined parameters serve as a foundation for improving the overall efficiency, sensitivity, and reliability of the sensor. In this section, a set of optimized parameters has been systematically identified and established through a detailed parametric optimization process. This method involves the careful adjustment of key design variables within specified ranges to achieve the most favorable outcomes in terms of sensor response and functionality. By analyzing the effects of individual parameters on the performance metrics—such as resonance frequency, quality factor, figure of merit, and detection limit—the design can be fine-tuned for optimal operation. The results obtained from this parametric study reveal how even small changes in specific variables can lead to significant improvements in performance. The selected optimized values represent a balance between competing requirements, ensuring that the sensor operates efficiently under targeted conditions. This approach not only streamlines the design process but also provides a more informed and data-driven foundation for future enhancements. Thus, the parametric optimization carried out here plays a crucial role in refining and validating the proposed sensor configuration.

**Fig. 5:**
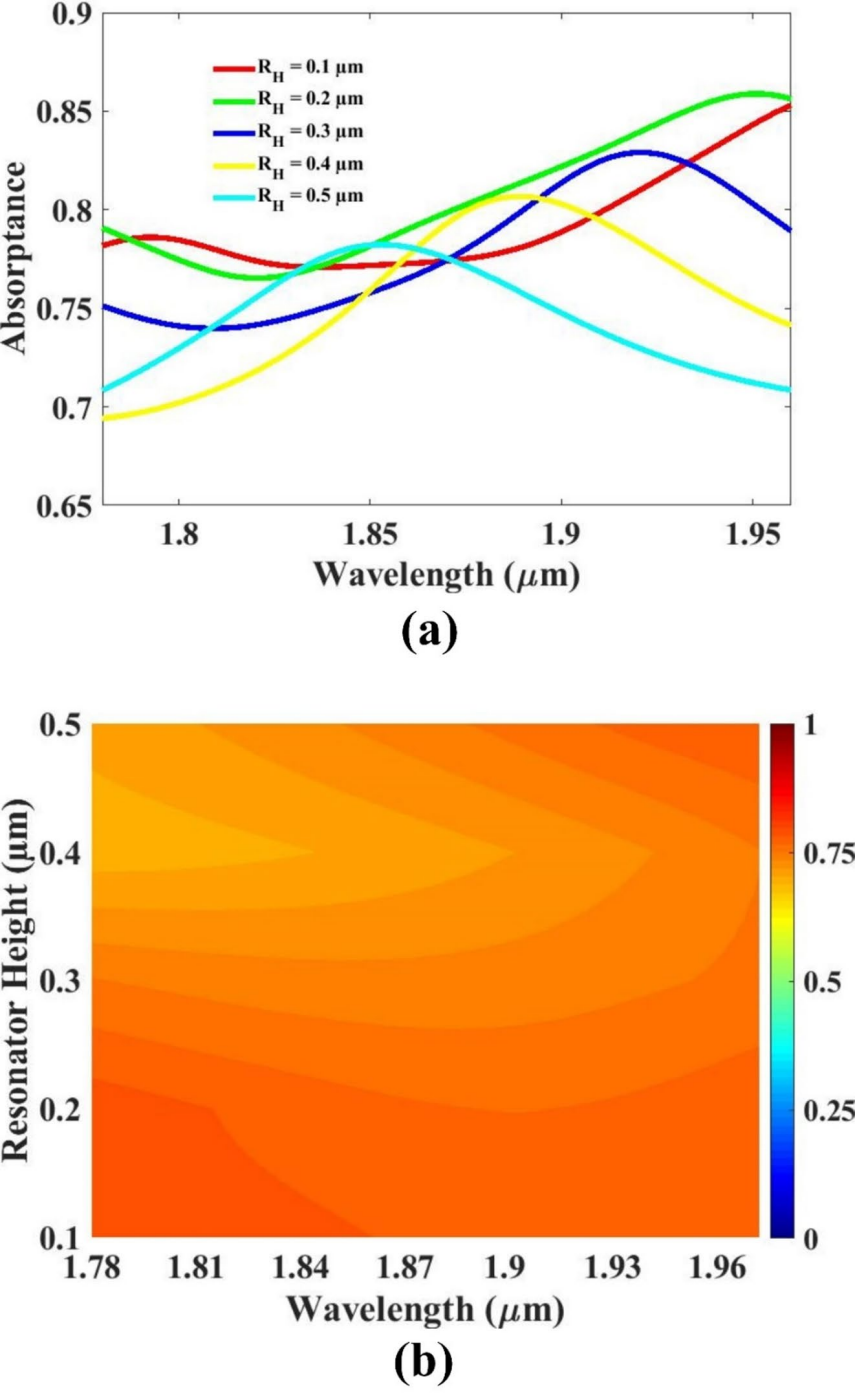
(**a**,**b**) Resonator thickness variation variation response.

**Fig. 6:**
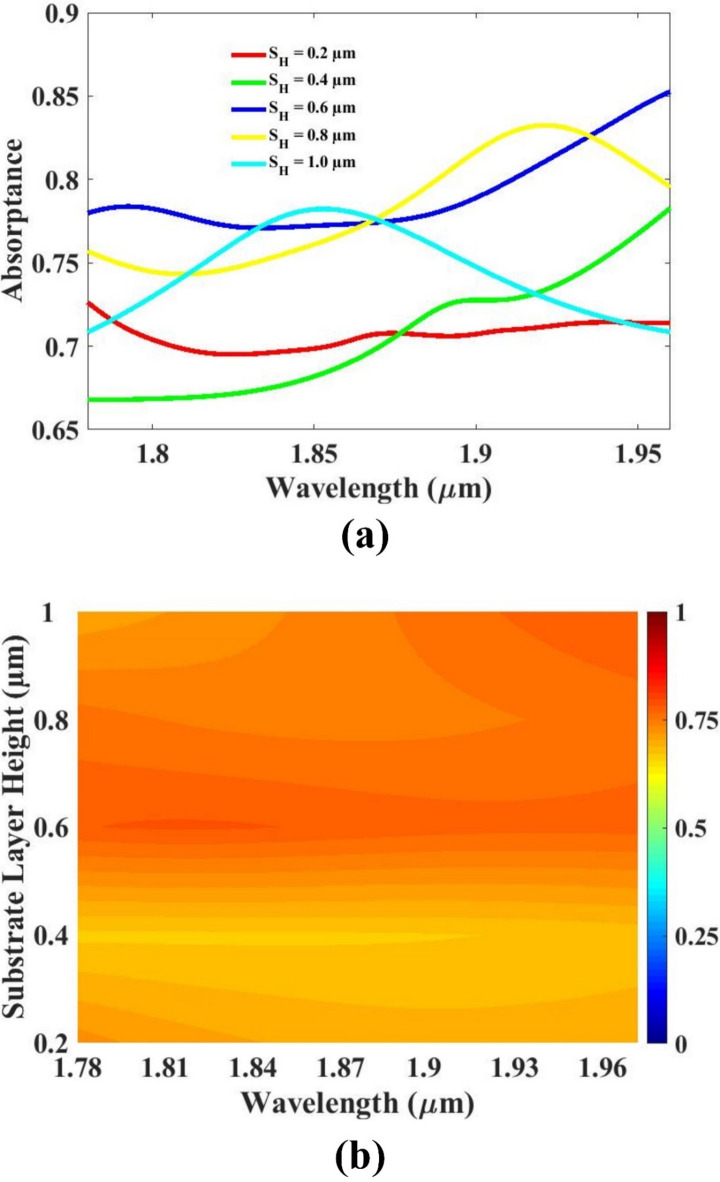
(**a**,**b**) Substrate thickness variation response.

**Fig. 7:**
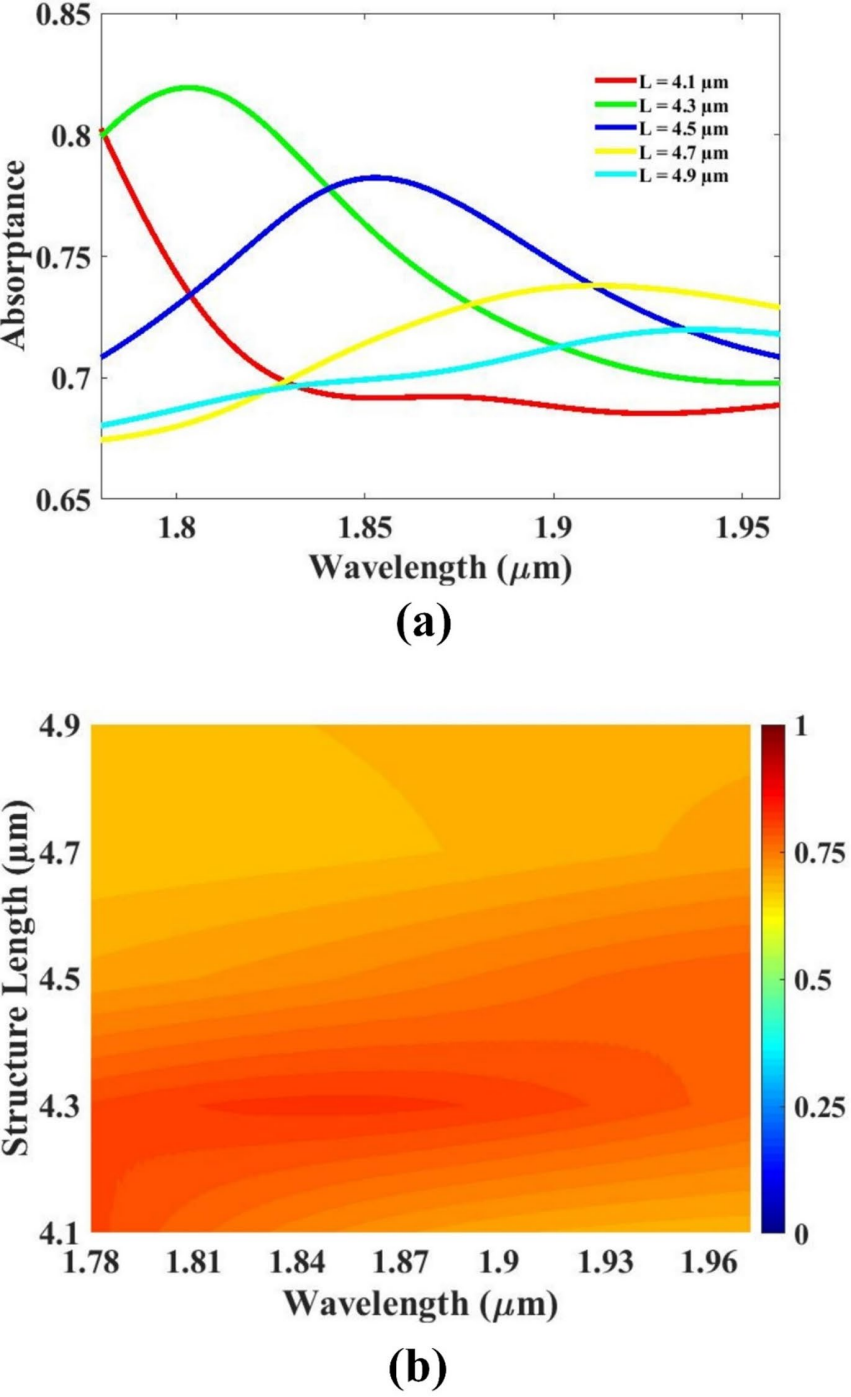
(**a**,**b**) Length variation response.

**Fig. 8:**
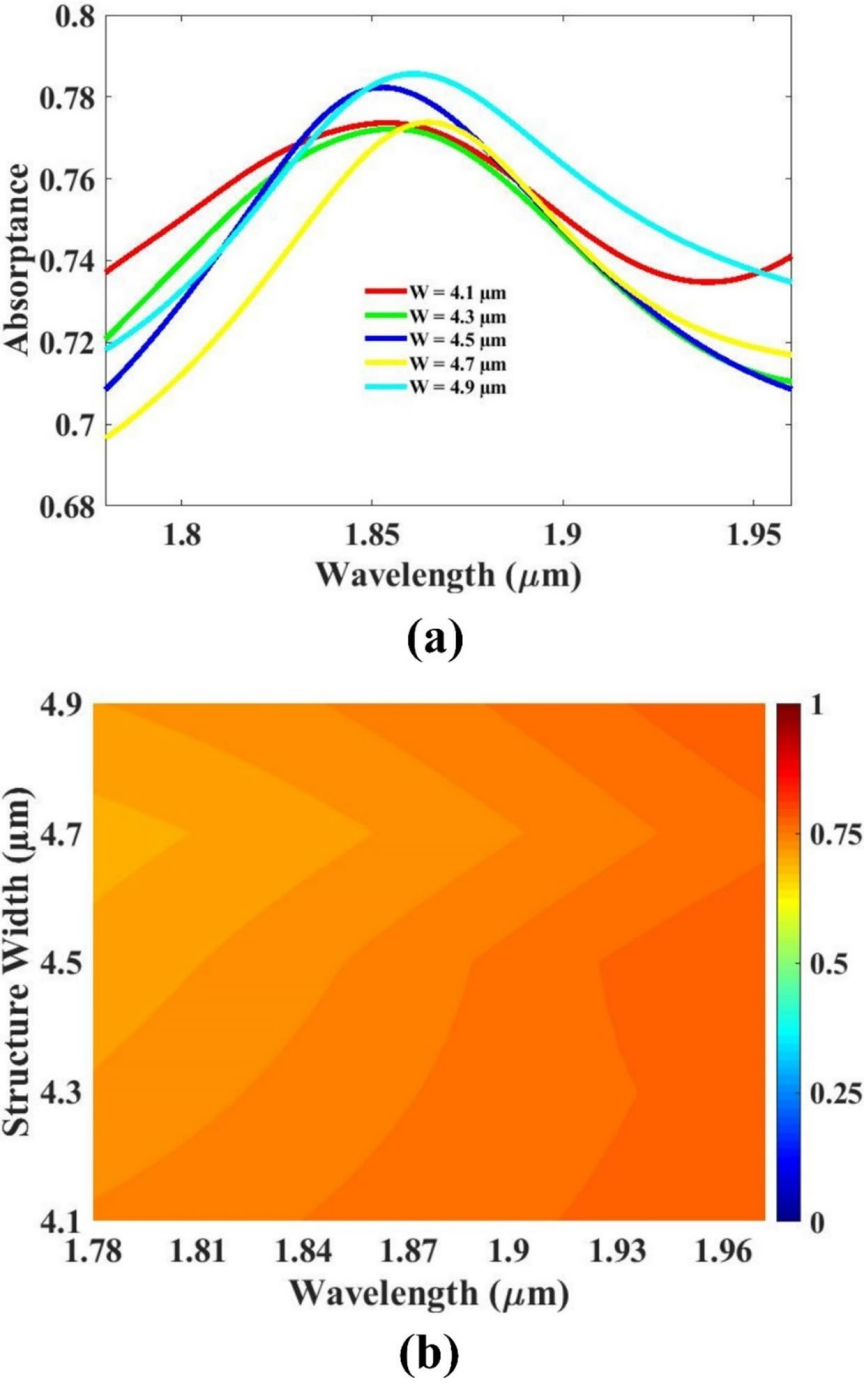
(**a**,**b**) Width variation response.

The thickness of the resonator, the thickness of the substrate, as well as the substrate’s length and width were systematically varied to study their influence on the overall sensor performance. By analyzing the sensor’s response to changes in these structural parameters, optimal values were determined to enhance efficiency and sensitivity. The impact of each parameter on key performance indicators was carefully evaluated through simulation and analysis. The resulting optimized configurations are presented and discussed in Figs. 5, 6, 7 and 8, offering valuable insights into how geometric modifications contribute to improved sensor design and functionality under specified operating conditions.

The thickness of the resonator was varied within the range of 0.1 μm to 0.5 μm to examine its influence on the sensor’s performance characteristics. Through this parametric investigation, it was observed that a thickness of 0.5 μm yielded the most favorable response, indicating it as the optimal value for this structural parameter. This specific thickness demonstrated improved resonance behavior and enhanced sensitivity, making it the most effective choice among the tested variations. The detailed response corresponding to these changes is illustrated in Fig. 5, which visually supports the selection of the 0.5 μm resonator thickness as the optimal configuration.

The thickness of the substrate was systematically varied from 0.2 μm to 1 μm to analyze its effect on the sensor’s performance and structural behavior. While a thinner substrate, such as 0.2 μm, is advantageous in terms of compact device dimensions and lightweight design, it was found to compromise the quality of the sensor’s response. Specifically, the reduced thickness led to performance limitations, including lower sensitivity and weaker resonance characteristics. Although minimizing the size is beneficial in certain applications, it should not come at the cost of sensor efficiency and accuracy.

In contrast, increasing the substrate thickness to 1 μm resulted in a significantly improved sensor response. The structural stability and interaction with the resonating elements were enhanced at this thickness, contributing to better performance metrics such as higher quality factor and stronger signal clarity. Therefore, after evaluating the trade-offs between miniaturization and functional performance, a substrate thickness of 1 μm was identified as the optimal choice for this design. This selection balances structural support with high-performance operation. The outcomes of this parameter variation, including the performance differences across the tested thickness range, are clearly illustrated and discussed in Fig. 6, validating the effectiveness of the chosen value.

To evaluate the impact of structural dimensions on sensor performance, the length of the substrate was varied in the range of 4.1 μm to 4.9 μm. The objective of this analysis was to identify a length that ensures a compact design while still delivering a strong and reliable response. A smaller substrate length is generally preferred for achieving a miniaturized sensor layout, which is particularly beneficial for applications requiring integration into limited spaces or portable systems. However, extremely short lengths were found to reduce the effectiveness of the sensor’s response, leading to diminished sensitivity and less pronounced resonance behavior. Through detailed simulation and performance assessment, it was determined that a length of 4.5 μm offers the best compromise between device miniaturization and high-performance operation. This value ensures a sufficiently compact design without sacrificing signal strength, quality factor, or sensitivity. Among the tested values, the 4.5 μm configuration produced the most favorable response, making it the optimal choice for the sensor layout. The variation in response across the tested range is clearly depicted in Fig. 7, which illustrates the advantages of selecting the 4.5 μm length in achieving both space efficiency and robust sensor functionality.

The width of the fabricated structure was systematically varied within the range of 4.1 micrometers to 4.9 micrometers to assess its effect on overall device performance. Among the different tested dimensions, the widths of 4.5 μm and 4.9 μm demonstrated notably better responses in terms of functional output and structural integrity. While both values yielded favorable results, the 4.5 μm configuration emerged as a more suitable choice when considering practical aspects such as component miniaturization, material consumption, and manufacturing cost-efficiency. Despite the slightly higher performance observed at 4.9 μm, the 4.5 μm width strikes a more optimal balance between performance and resource utilization. As a result, it has been selected as the optimized width dimension for subsequent design considerations and fabrication processes. This selection allows for a compact structural footprint, which is particularly beneficial in applications requiring high device density or cost-effective production. Moreover, opting for the smaller width facilitates better scalability in integrated systems without compromising the required operational standards. In conclusion, although several widths were evaluated, the 4.5 μm structure was identified as the best compromise between efficiency, performance, and practical manufacturing benefits, thereby making it the finalized choice for the optimized design parameter in this study. The response is investigated in Fig. 8.

**Fig. 9:**
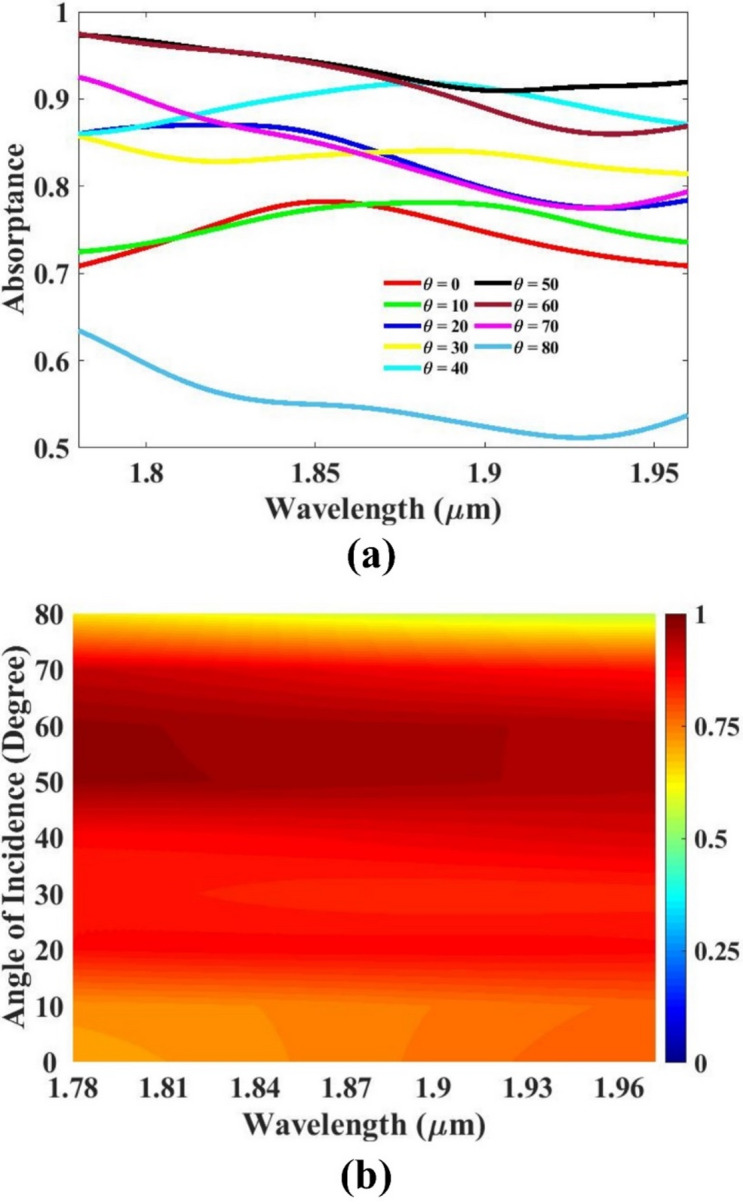
(**a**,**b**) Angle of incidence variation response.

The angle performance of the sensor design is anlayzed. This parameter plays a significant effect in showing how the sensor interacts with incoming signals, particularly in optical or wave-based systems. The sensor’s responsiveness exhibited noticeable improvement, suggesting that broader angles contribute positively to its functionality. The analysis revealed that the sensor maintains a consistent and effective performance over a wide range of incidence angles, specifically from 0 degrees up to 70 degrees. This operational range is illustrated in Fig. 9, where the results highlight the sensor’s adaptability and robustness across varying directional inputs. The ability of the sensor to perform reliably at wider angles is an important advantage, as it enhances the versatility of the device in practical applications. Systems that operate under dynamic conditions often encounter signals from multiple directions, so a design capable of sustaining performance across such a broad angular range is highly beneficial. The results confirm that the sensor design remains efficient even when the angle of incidence is significantly altered, establishing a key performance benchmark. Thus, the sensor demonstrates a strong angular response profile, supporting its suitability for diverse real-world deployment scenarios.

### Electric field intensity

**Fig. 10:**
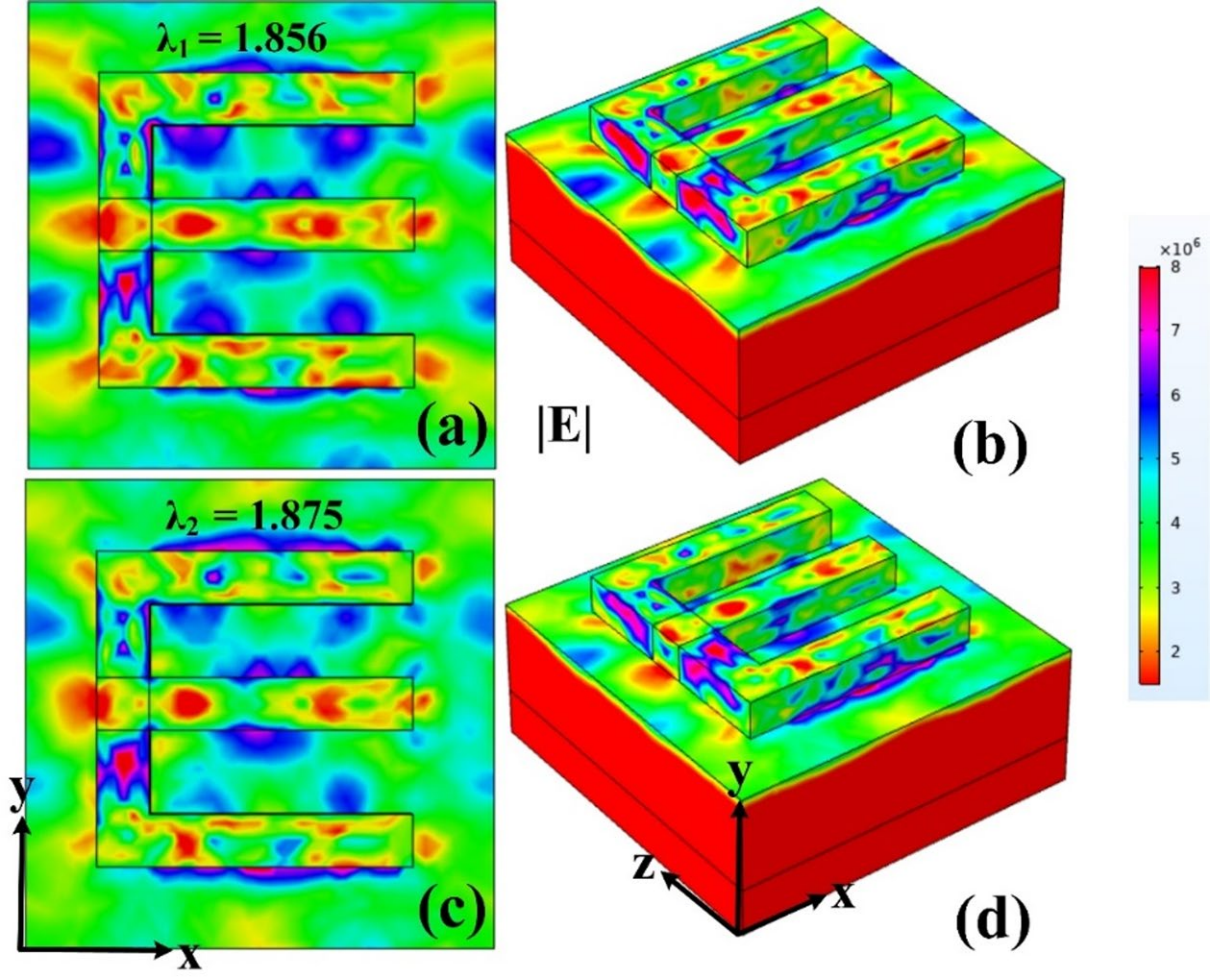
(**a–d**) Electric field response. Showing fieldsn for two wavelengths 1.856 μm and 1.875 μm.

The E-field was presented to examine how absorption characteristics influence the field distribution at each wavelength. This comparative study aimed to correlate variations in the electromagnetic field with corresponding absorption behavior. The simulation results indicate that the field intensity behaves consistently with the absorption trends, confirming a strong relationship between the two parameters. As illustrated in Fig. 10, the field patterns closely align with the absorption profiles at both wavelengths, validating the accuracy and effectiveness of the model. These observations demonstrate that changes in wavelength significantly impact the electric field distribution, with higher absorption corresponding to stronger field confinement. This alignment reinforces the understanding of how material and structural properties interact with incident wavelengths, offering insights into optimizing the design for enhanced performance. The matching results between field intensity and absorption not only affirm the reliability of the model but also provide a deeper understanding of light-matter interactions within the structure. Overall, the electric field responses observed for the two wavelengths support the conclusion that the structure effectively manipulates electromagnetic behavior in agreement with its absorption efficiency, strengthening the design’s relevance for wavelength-selective or absorption-based applications.

A detailed comparative analysis between the proposed work and other relevant studies previously published in the literature is provided in Table 1. This comparison serves to highlight the strengths, improvements, and unique features of the current design relative to existing solutions in the same domain. By evaluating key performance parameters such as efficiency, structural dimensions, fabrication complexity, and material usage, the table allows for a clear understanding of where the proposed approach stands in relation to similar research efforts. The studies selected for comparison were chosen based on their methodological similarities, functional objectives, and relevance to the field. This side-by-side evaluation brings out the distinct advantages of the current design, including enhanced performance metrics and practical benefits such as reduced size, better material compatibility, and cost-effectiveness. Additionally, the comparison underscores the innovations introduced in the proposed work, such as optimized geometric parameters and improved absorption or sensitivity levels, depending on the application. Table 1 thus serves as a comprehensive summary that emphasizes the progress made through this research, illustrating measurable gains over previously reported techniques. This structured assessment not only validates the proposed design’s effectiveness but also establishes its potential for further development and practical implementation in real-world applications.

**Table 1:** Comparison.

Design	Sensitivity (nm/RIU)	Quality factor (Q)	Figure of merit (FOM)	Detection limit (DL)
^38^	253	–	–	–
^39^	349	–	–	–
^40^	1000	5166	2.94	0.04
^41^	74.5	19.82	–	–
^42^	153	–	–	–
^43^	161	–	–	–
^44^	690	2500	1400	–
^45^	200	3493	222	–
^46^	1000	–	–	–
^47^	500	–	–	–
^48^	1100	–	3.832	0.391
^49^	387	–	–	–
^50^	596	–	–	–
^51^	900	190	105	0.00001
^52^	400	–	–	–
^53^	546.72	2066.44	–	1.44
^54^	1000	–	–	–
^55^	1400	–	–	–
^56^	306.25	5000	103	–
^57^	–	262	–	0.002
^58^	359	–	–	–
Proposed blood cancer design	1430	125	121	0.044

## Machine learning optimization

The current sensor analysis, based on the variation of output parameters, is demonstrated through machine learning results. Using the linear regression method, the resonator height, substrate height, and design width of the sensor have been analyzed. The machine learning outputs are evaluated in terms of R^2^ values and the minimum root mean square error (RMSE), with a test size of 0.25.

**Fig. 11:**
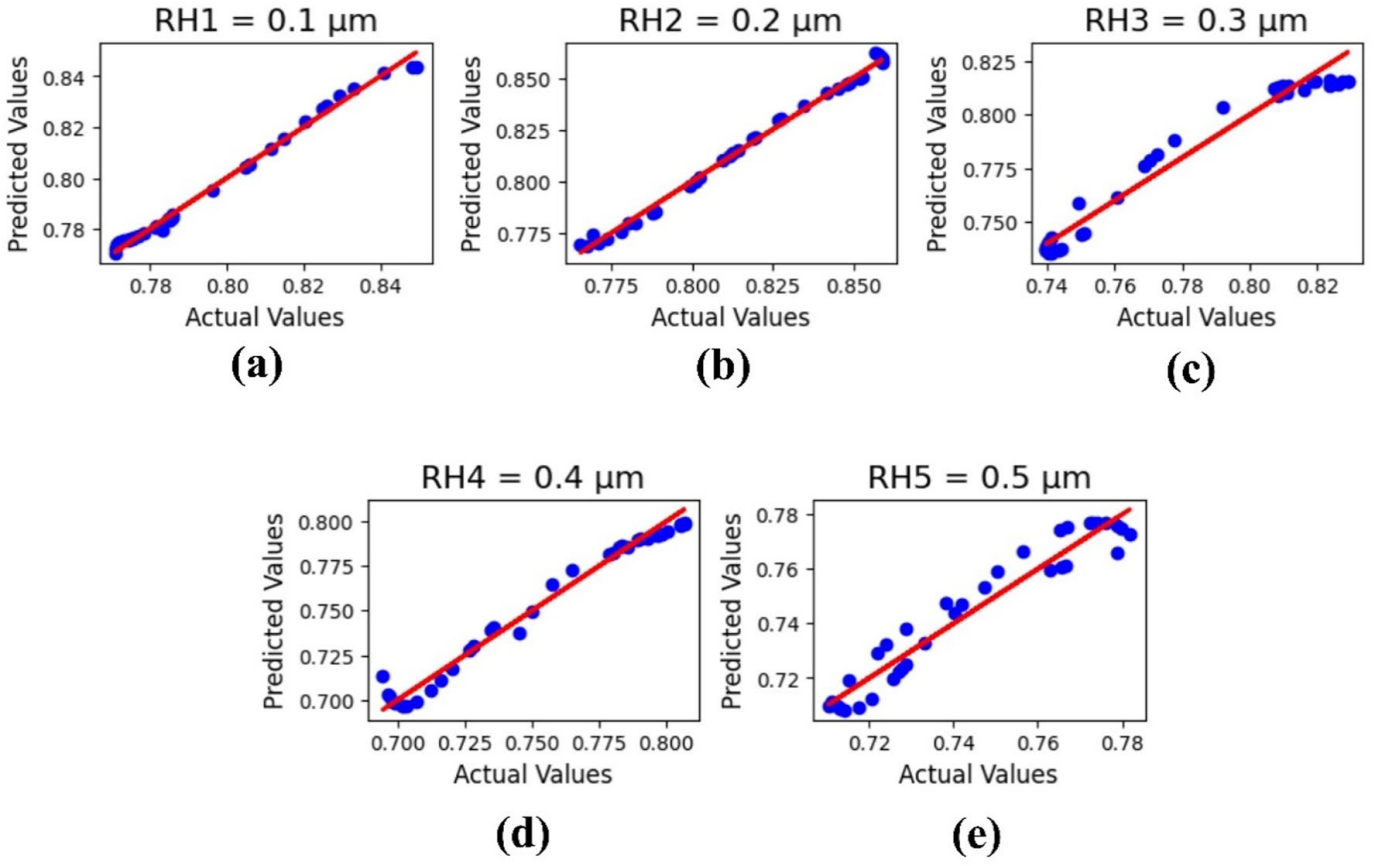
ML outputs for resonator height variations in µm: (**a**) 0.1, (**b**) 0.2, (**c**) 0.3, (**d**) 0.4, and (**e**) 0.5.

Figure 11 shows the machine learning output analysis based on the variation of resonator height ‘RH’ from 0.1 to 0.5 μm. The ML results are presented as R^2^ values: 0.928303838, 0.9514725064, 0.96123380987, 0.979164224751, and 0.933993509951, with a MSE value of 4.294081206193 × 10⁻⁶.

**Fig. 12:**
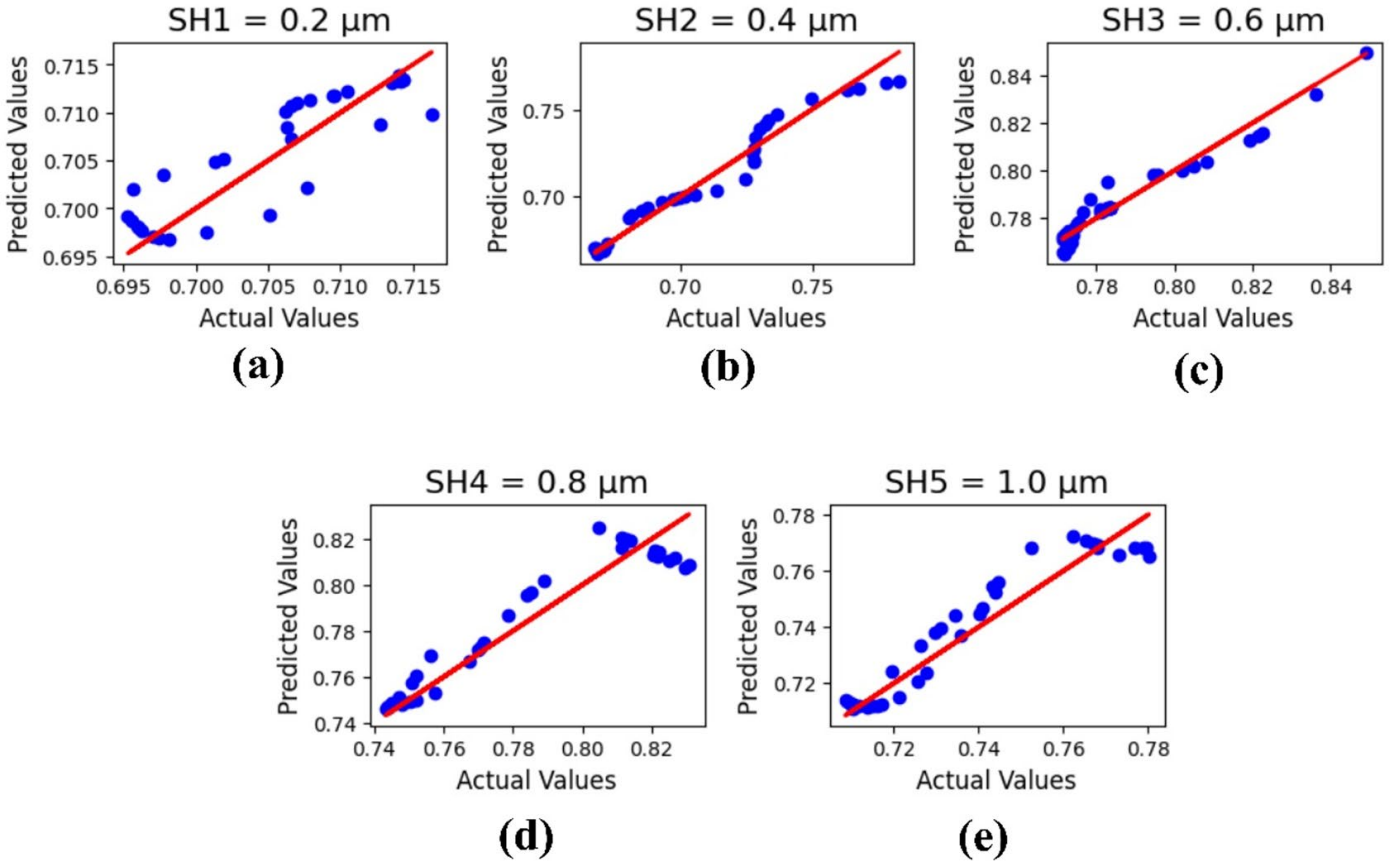
ML outputs for substrate height variations in µm: (**a**) 0.2, (**b**) 0.4, (**c**) 0.6, (**d**) 0.8, and (**e**) 1.0.

Figure 12 shows the machine learning output analysis based on the variation of substrate height ‘SH’ from 0.2 to 1.0 μm. The ML results are presented as R^2^ values: 0.81161760799, 0.9619364920148, 0.94928347521, 0.914463361423, and 0.91116242007, with MSE value 1.9524528860013 × 10⁻^5^.

**Fig. 13:**
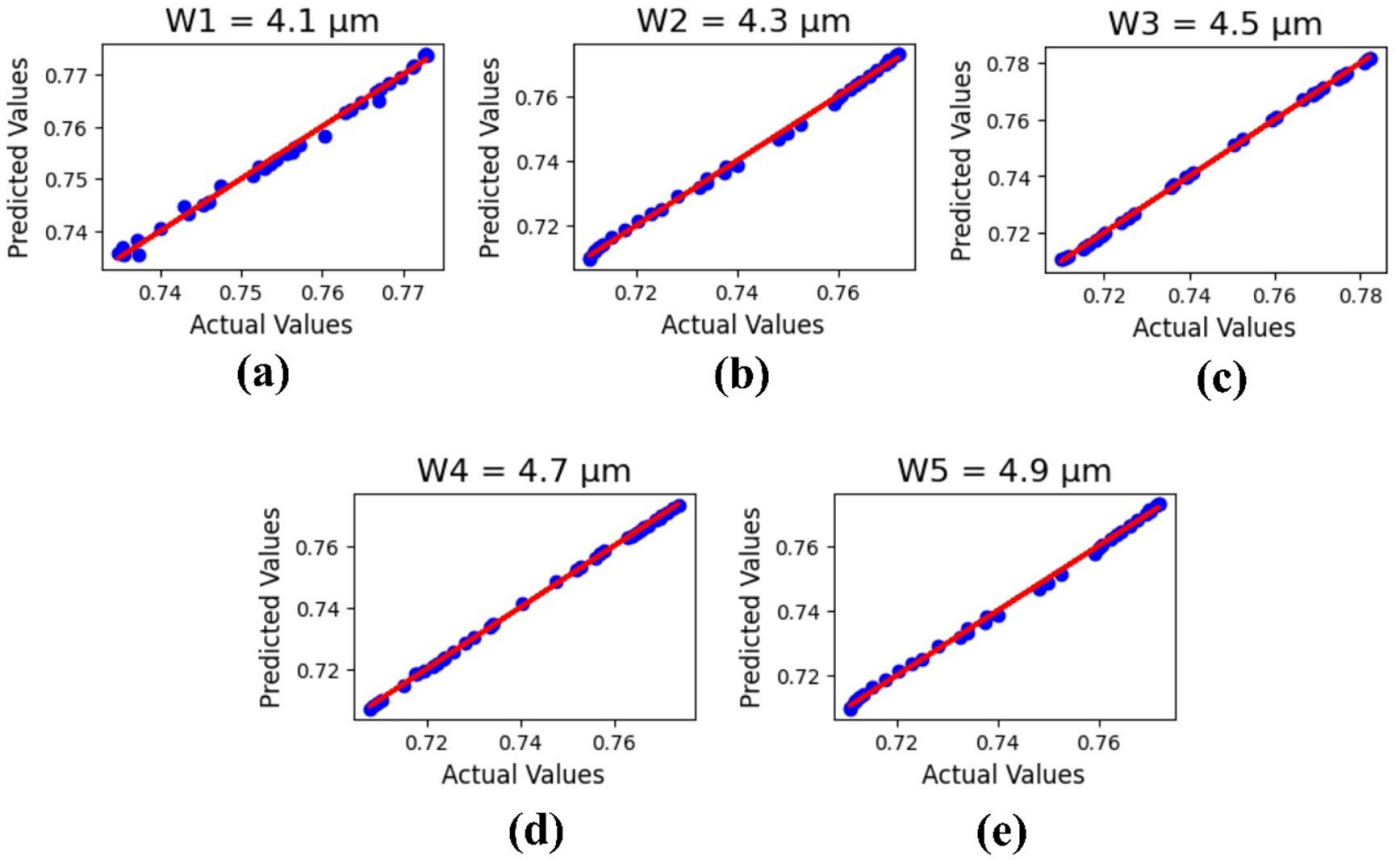
ML outputs for sensor design width variations in µm: (**a**) 4.1, (**b**) 4.3, (**c**) 4.5, (**d**) 4.7, and (**e**) 4.9.

Figure 13 shows the machine learning output analysis based on the variation of sensor design width ‘W’ from 4.1 to 4.9 μm. The ML results are presented as R^2^ values: 0.99487475577, 0.9984742441422, 0.9925667895314, 0.9995565860592, and 0.998775642218, with a MSE value 2.2389093397 × 10⁻^7^.

## Conclusion

In conclusion, the sensor design demonstrates excellent performance based on comprehensive parametric analysis. A high sensitivity of 1430 nm/RIU confirms the sensor’s strong capability to detect even minute changes in the refractive index, making it highly suitable for applications in chemical and biological sensing. The sharp and well-defined resonance achieved in the design is supported by a Q-factor of 125, indicating a relatively narrow resonance peak and stable signal output. This enhances the sensor’s accuracy and resolution, which are critical for precise detection tasks. Furthermore, the Figure of Merit (FOM) value of 121 highlights the sensor’s efficiency in combining sensitivity with spectral resolution, reflecting its ability to differentiate between closely spaced refractive index changes. A low Detection Limit (DL) of 0.044 RIU signifies the design’s ability to identify extremely small variations in the refractive index, further emphasizing its suitability for ultra-sensitive sensing environments. The overall performance, derived through detailed parametric optimization, showcases a balanced and highly effective design. The combination of high sensitivity, strong Q-factor, excellent FOM, and low DL affirms that this sensor configuration holds significant promise for real-world applications requiring precision and reliability. Future work may explore its integration into compact and scalable sensing platforms.

## Data Availability

The data supporting the findings in this work are available from the corresponding author with reasonable request.
